# Abnormal Dynamic Reconstruction of Overlapping Communities in Schizophrenia Patients

**DOI:** 10.3390/brainsci14080783

**Published:** 2024-08-01

**Authors:** Yuxiang Guo, Xubin Wu, Yumeng Sun, Yanqing Dong, Jie Sun, Zize Song, Jie Xiang, Xiaohong Cui

**Affiliations:** 1 School of Software, Taiyuan University of Technology, No.209, University Street, Jinzhong 030600, China; sxtygyx@163.com; 2 College of Computer Science and Technology (College of Data Science), Taiyuan University of Technology, No.209, University Street, Jinzhong 030600, China; wuxubin0066@link.tyut.edu.cn (X.W.); m15735216932@163.com (Y.S.); d15110464375@163.com (Y.D.); sunjie0086@link.tyut.edu.cn (J.S.); 15110302796@163.com (Z.S.); xiangjie@tyut.edu.cn (J.X.)

**Keywords:** dynamic overlapping community algorithm, fMRI, dynamic brain network, schizophrenia, community restructuring

## Abstract

Objective: This study aims to explore the changes in dynamic overlapping communities in the brains of schizophrenia (SZ) patients and further investigate the dynamic restructuring patterns of overlapping communities in SZ patients. Materials and Methods: A total of 43 SZ patients and 49 normal controls (NC) were selected for resting-state functional MRI (rs-fMRI) scans. Dynamic functional connectivity analysis was conducted separately on SZ patients and NC using rs-fMRI and Jackknife Correlation techniques to construct dynamic brain network models. Based on these models, a dynamic overlapping community detection method was utilized to explore the abnormal overlapping community structure in SZ patients using evaluation metrics such as the structural stability of overlapping communities, nodes’ functional diversity, and activity level of overlapping communities. Results: The stability of communities in SZ patients showed a decreasing trend. The changes in the overlapping community structure of SZ patients may be related to a decrease in the diversity of overlapping node functions. Additionally, compared to the NC group, the activity level of overlapping communities of SZ patients was significantly reduced. Conclusion: The structure or organization of the brain functional network in SZ patients is abnormal or disrupted, and the activity of the brain network in information processing and transmission is weakened in SZ patients.

## 1. Introduction

Schizophrenia (SZ) is a complex psychiatric disorder that usually occurs in adolescence or adulthood and is associated with abnormal integration between distal brain regions [1,2]. The disorder is considered to be a chronic and diverse genetic disorder with abnormal perceptual, emotional, and brain connectivity, clinically characterized by emotional and cognitive dysfunction with hallucinations, delusions, and other symptoms [3,4,5]. Therefore, SZ is increasingly recognized as a disorder caused by brain network dysfunction [6,7]. SZ, as defined by *The Diagnostic and Statistical Manual of Mental Disorders* (DSM), is characterized by a specific set of symptoms that can be categorized into positive and negative symptoms [8]. Patients with SZ have exhibited modifications in brain networks, as evidenced by numerous fMRI studies. Moreover, alterations in neurotransmitters and neurochemical levels have also been observed [9], which are hypothesized to be linked to these network changes. Several studies have associated the aforementioned alterations with brain network modifications using resting-state functional MRI (rsfMRI) [10].

A community is an entity formed by a set of nodes and their edges that are tightly connected, and community detection is an important issue in network science, which aims to detect the presence of communities in a network by utilizing the principles of tight connectivity between nodes and sparse connectivity of nodes between communities [11]. The traditional non-overlapping community model assumes that brain regions are strictly divided into different functional communities, that each brain region belongs to only one specific functional community, and that the communities have clear boundaries and do not overlap each other. For example, Wang et al. found significant abnormalities in the community structure of Alzheimer’s disease patients in terms of integration and segregation mechanisms using Leuven’s community detection algorithm [12]. Ding et al.’s study found altered community structure in the brains of ADHD patients and significant abnormalities in the dynamic reconfiguration and flexibility of the patients’ brain communities [13]. Lerman-Sinkoff et al. utilized the Infomap’s community detection method to study the community structure of SZ patients and found that the patients’ community structure was found to be abnormal in the sensorimotor, auditory, default, attention, limbic/paralimbic, and subcortical networks, with the most significant changes occurring in the bilateral thalamus [14]. SZ is widely recognized for its impact on brain network dysfunction, affecting various aspects of cognitive and emotional processing. This dysfunction is thought to underlie the complex symptoms experienced by individuals with SZ. Building on this foundational understanding, our study specifically focuses on the role of overlapping communities within these networks. By examining the dynamic interplay of these communities, we aim to shed light on the intricate mechanisms that contribute to the pathophysiology of SZ.

With the rapid development of neuroimaging techniques and data analysis methods, more and more studies have shown that many brain regions may participate in multiple functional networks at the same time while performing different functional tasks, forming overlapping community structures [15,16]. The overlapping community structure means that a brain region can belong to multiple functional communities at the same time, and this structural flexibility provides the brain with higher information processing efficiency and adaptability [17,18,19]. By participating in multiple functional networks, brain regions are able to form more complex and enriched connectivity patterns among themselves, thus supporting broader information transfer and integration [20]. In addition, the overlapping community structure can facilitate the interaction and coordination between different functional networks, which helps the brain to realize more complex and fine-grained cognitive functions [21,22,23,24,25].

However, despite the progress made in detecting community structures in brain networks, existing research has primarily focused on detecting non-overlapping communities, overlooking an important issue: the situation where a brain region belongs to multiple communities simultaneously. The existence of this scenario suggests that the current non-overlapping community detection methods have certain limitations in fully reflecting the true complexity of brain functional networks. Non-overlapping community detection methods often classify each brain region into a single community, leading to information loss and simplification. Especially for functionally complex and densely connected brain regions, they may play multiple roles in different functional networks, but this diversity is often not fully captured by non-overlapping community detection methods. Therefore, researchers have begun to focus on the study of overlapping communities to more comprehensively understand the structure and function of brain networks, which has become an important direction in current brain network research.

**Figure 1:**
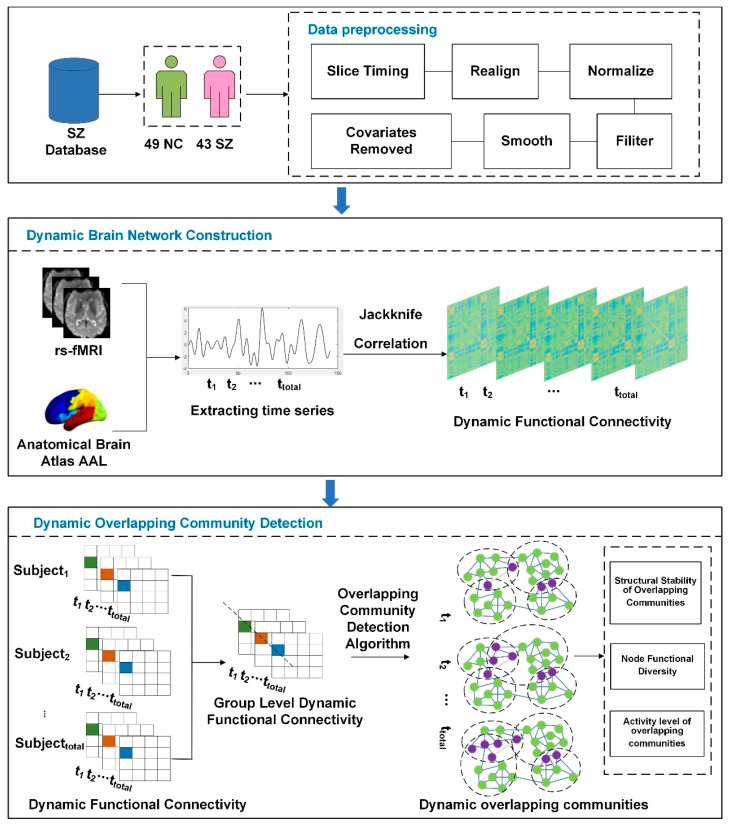
Schematic overview of the analysis strategy. First, the standard brain profile in the AAL template was divided into 90 separate brain regions. Each region represents a node in the study network. Functional connectivity at each time point was then estimated using the Jackknife Correlation method. Next, dynamic functional connectivity was group leveled. By employing the matrix decomposition technique, the group-level dynamic functional connectivity matrix after group leveling was decomposed into two matrices. One of the matrices preserved the membership information of the community, while the other traced the dynamic changes of the community over time. The structure of overlapping communities of patients was analyzed through a series of metrics, including the structural stability of overlapping communities, nodes’ functional diversity, and activity level of overlapping communities.

Therefore, this study extends the overlapping community detection method for static networks to consider temporal information to detect overlapping communities in dynamic networks and proposes a dynamic overlapping community detection method based on matrix decomposition to explore the differences in overlapping community structure between SZ patients and normal controls (NC), which provides new perspectives and research directions to explore the principles of functional organization of the SZ brain (Figure 1).

## 2. Materials and Methods

### 2.1. Participants

Data for this study were obtained through a public database (https://openfmri.org/dataset/ds000030/ (accessed on 18 October 2021)). After data screening, some of the data were found to be non-compliant with the experimental requirements, which may have affected the results of the experiment, and were not selected as subjects. The exclusion criteria were as follows: during processing, the Mean FD Jenkinson in the HeadMotion.csv file was used as the criterion to exclude the subject data according to the threshold value greater than 0.2. There was a total of 92 subjects with resting-state functional magnetic resonance imaging data, including 43 SZ patients and 49 NC subjects. There were no significant differences in age and sex (*t*-test [23]) between NC and SZ patients. Also, SZ patients participated in the Scale for Assessment of Positive Symptoms (SAPS) [24] for adults. Higher SAPS scores indicate a higher severity of positive symptoms associated with SZ. Specific demographic characteristics are shown in Table 1.

### 2.2. Imaging Acquisition and Preprocessing

Neuroimaging data of both SZ patients and NC were acquired by functional MRI scanning on a 3T Siemens Trio scanner. During the acquisition process, soft foam and earplugs were used to immobilize the subject’s head, thus reducing noise during the scanning process. At the same time, the subjects were asked to remain relaxed with their eyes open and to avoid making movements or engaging in intentional thinking activities. fMRI data were acquired from a T2-weighted planar echo-imaging (EPI) sequence with the following parameters: slice thickness = 4 mm, number of slice layers = 34, echo time (TE) = 30 ms, flip angle = 90°, field of view (FOV) = 192 mm, repetition time (TR) = 2 s, and resolution = 64 × 64. The resting-state functional magnetic resonance imaging data scan lasted a total of 304 s, and 152 time points were acquired.

Preprocessing of resting-state functional MRI data was performed on the toolbox DPABI (Data Processing & Analysis for Brain Imaging, http://rfmri.org/dpabi (accessed on 15 October 2021) [26]. The subjects’ ability to adapt to the environment as well as the equalization of the signals can affect the results of the experiment. Therefore, for each subject’s resting-state fMRI data, the first 10 time points were removed, leaving 142 time points. The data were then preprocessed as follows:

(1) A total of 34 slices of fMRI data were used, and the scan times of all layers were corrected to the same moment using an interpolation algorithm. (2) Rigid transformation was used to perform head movement correction, using the first layer image as the target image, aligning all images with the target image, and excluding subjects with a rotation angle greater than 2 degrees and a translation greater than 2 mm. (3) Spatial normalization was performed [27]. (4) The images were band-pass filtered in the range of 0.01–0.10 Hz [28]. (5) Spatial smoothing was performed using a Gaussian kernel convolution completion of 6 mm Full Width at Half Maximum (FWHM) [29]. (6) The removal of covariates was employed to reduce the effect of non-neuronal BOLD signals using linear regression on the interfering variables.

The brain was also divided into 116 regions using the AAL template [30] (detailed information is provided in Table A1), and the remaining time series were extracted for each voxel. In this study, only the first 90 brain regions were studied, and the last 26 cerebellar regions were not studied. The AAL template divides the brain regions into five functional networks [31,32]: the Sensory-Motor Network (Sensorimotor Network, SMN), Visual Network (Visual Network, VN), Attention Network (Attention Network, AN), Default Network (Default Mode Network, DMN) and Limbic/Paralimbic and Subcortical Network (LSN).

### 2.3. Construction of Dynamic Brain Networks

Meanwhile, the Jackknife Correlation (JC) method [33,34,35,36] was used in this study to estimate the functional connectivity at a single time point. When the JC method was applied to the single time point covariance estimation of signals *x* and *y* at time point *t*, the Pearson correlation between the two signals was calculated using all time points in x and y except $x_{t}$ and $y_{t}$. The JC of two Blood Oxygenation Level Dependent (BOLD) signal time series x and y at time point t is shown in Equation (1):

(1) $$\begin{array}{c} JC_{t}=-\left(\frac{\Sigma_{t_{i}}^{t_{total}}\left(x_{t_{i}}-\bar{x_{t}}\right)\left(y_{t_{i}}-\bar{y_{t}}\right)}{\Sigma_{t_{i}}^{t_{total}}{\left(x_{t_{i}}-\bar{x_{t}}\right)}^{2}\Sigma_{t_{i}}^{t_{total}}{\left(y_{t_{i}}-\bar{y_{t}}\right)}^{2}}\right),\;t_{i}\neq t \end{array}$$

where $t_{total}$ is the total number of time points. The $\bar{x_{t}}$ and $\bar{y_{t}}$ are the expected values excluding the data at time point t:

(2) $$\begin{array}{c} \bar{x_{t}}=\frac{1}{t_{total}-1}\sum_{t_{i}}^{t_{total}}x_{t_{i}},\;t_{i}\neq t \end{array}$$

(3) $$\begin{array}{c} \bar{y_{t}}=\frac{1}{t_{total}-1}\sum_{t_{i}}^{t_{total}}y_{t_{i}},\;t_{i}\neq t \end{array}$$

The Equation (1) shows how to calculate this correlation. In simple terms, it is a mathematical way to tell us whether the activities of two brain regions are still closely related after excluding data from a specific moment. Notably, previous studies have shown that the JC method performs better in tracking covariance time changes than other methods. Here, each JC value was normalized, and the standardized JC method was unaffected by the underlying static properties. The JC method estimated paired functional brain connections at each time point. This information could be used to investigate the dynamic characteristics of a given network and the interactions between different nodes within it.

### 2.4. Dynamic Functional Connectivity at the Group Level

For the SZ patient group and the NC group, each group obtained S×T connectivity matrices of size N×N, where S is the total number of subjects, T is the total number of time points, and N is the number of brain regions. Prior to the overlapping community test, this study introduced a method of constructing group-level dynamic functional networks [37] to represent the characteristics of SZ patients and NC, respectively. The group-level dynamic functional network reflects the common characteristics of the entire group rather than those of individual subjects, thus better capturing common patterns and overall characteristics within the group. The method captures the connectivity between ROI nodes in all time points and selects the set of feature vectors closest to the group level from all subjects. For each pair of brain regions i and j in the dynamic functional network of each subject, cross-time point feature vectors combining the connectivity weights of all time points were extracted. To compute group-level dynamic functional connectivity for each pair of ROI nodes i and j, the relationship of feature vectors across subjects was modeled, and the vector closest to the other feature vectors was selected as the representative vector. The above process was repeated for each pair of brain regions, and the group-level dynamic brain network was constructed. The specific iterative process pseudo-code of the method is shown below (Algorithm 1).
**Algorithm 1.** Group-level dynamic brain network construction algorithm**Input:**Functionally connected network of a total of S subjects with a four-dimensional matrix W of size S ∗ N ∗ N ∗ T
**Process:***begin* **for** *i = 1 to N* **do**  **for** *j = 1 to N* **do**    *Initialization Vector ds = [];*    **for** *s1 = 1 to S* **do**      *Initializing Variables d = 0;*      **for** *s2 = 1 to S* **do**        *d = d +*$∥W\left[s1,:,i,j\right]-W\left[s2,:,i,j\right]{∥}_{2}$*;*      **end**      *ds = [ds, d];*    **end**    *Initialize the variable;* *    index= Index of the smallest value in vector ds;* *    G[:,i,j] = W[index,:,i,j];*  **end****end** **end** **Output:** Group-level dynamic brain network with a three-dimensional matrix F of size N ∗ N ∗ T


### 2.5. Overlapping Community Detection in Dynamic Brain Networks

Given the computed group-level dynamic functional connectivity matrix, the functional connectivity matrix for each time point is $F^{i}\in R^{n*n}\;(i=1,\;\ldots ,\;z)$, where n and z denote the number of nodes and time points, respectively. The dynamic overlapping community detection method detects the community structure of the brain network by optimizing the following objective function. The dynamic overlapping community detection method is shown in Equation (4):

(4) $$\begin{array}{c} \begin{array}{c} \underset{C,T\geq 0}{\min}\sum_{i=1}^{N}\left|\right|{F^{i}-{CZ}^{i}C^{T}||}_{F}^{2}+\beta {\left\|C\right\|}_{1}\\ s.t.\forall j:max\left(c_{j}\right)=1,j=1,\ldots ,k \end{array} \end{array}$$

where k is the number of detected communities. The column vector $c_{j}$ is the membership vector of the jth community. Also, for each time point, specific time-varying dynamic information is retained in the vector $z^{i}=\left(z_{1}^{i},\;z_{2}^{i},\;\ldots ,\;z_{k}^{i}\right)\in R^{k}$, where $c_{j}^{i}$ denotes the intensity of the jth community in the ith time point. C and $Z^{i}$ are non-negative, ${\left\|.\right\|}_{F}$ denotes the Frobenius paradigm. The regularization parameter β controls the sparsity of the detected communities. With the sparsity constraint, the most relevant nodes can be forced to be retained. The functional connectivity matrix is calculated as shown in Equation (5):

(5) $$\begin{array}{c} F^{i}\;\approx {\;z}_{1}^{i}c_{1}c_{1}^{T}+{\;z}_{2}^{i}c_{2}c_{2}^{T}+\ldots +z_{k}^{i}c_{k}c_{k}^{T} \end{array}$$

The optimization problem for $Z^{i}$ can be solved using the multiplicative update algorithm. For the optimization problem of C, this study used the projected gradient descent method to solve the problem. The pseudo-code of the algorithm is shown below (Algorithm 2):
**Algorithm 2.** Iterative process of dynamic overlapping community algorithm for brain networks based on matrix decomposition**Iutput:** Matrix $F^{i}$ **Parameter value selection:**The values of sparsity β and number of communities k are selected by grid search using a cross-validation algorithm**Process:**(1) Initialization: For each time point, randomly initialize $Z^{i}(i=1,\ldots ,z)$ as a non-negative diagonal moment of size k ∗ k; Randomly initialize C to a non-negative matrix of size n ∗ k; Normalize the column vector $c_{j}(j=1,\ldots ,k)$ of C;(2) Iterate until convergence: Fixed C; Update $Z^{i}(i=1,\ldots ,z)$ in each time point using the multiplicative update rule;Fixed $Z^{i}$; Update C by using projected gradient descent;Set all negative values in C to zero;Renormalize the column vector $c_{j}(j=1,\ldots ,k)$ of C; **Output:** Matrix C and matrix $Z^{i}$


Furthermore, a grid search approach coupled with 2-fold cross-validation was employed to optimize the parameters k and β. Specifically, k was varied from 2 to 20, while β ranged from 0 to 1 in increments of 0.1. The selection of the k range was informed by prior research studies, ensuring a comprehensive exploration of the parameter space. In addition, for each calculation, 20 random initialization runs were performed to select the best result for the minimum value of the objective function.

In summary, in order to obtain the public community structure at multiple time points, this study simultaneously decomposed the functional connectivity matrices at all time points instead of using the commonly used static functional connectivity matrices as input. The direct benefit brought by the present algorithm is to retain specific time-varying dynamic information, based on which the diagonal weight matrix $Z^{i}$ is further introduced to preserve the differences between time points. In addition, an important difference between the present dynamic overlapping community detection method and other matrix decomposition methods is that the non-negative constraints enable the dynamic overlapping community detection for the partially represented brain network functional connectivity, which can enhance the interpretability of the results.

### 2.6. Overlapping Community Metrics for Dynamic Brain Networks

(1) Structural Stability of Overlapping Communities

The aim of this study was to investigate whether there were differences in the optimal community vectors between SZ patients and NC through overlapping community structure stability analysis. Given that dynamic overlapping community detection methods may produce different community structures in each run due to different initialization conditions, this study paid particular attention to cross-run stability to ensure that it could more realistically reflect the underlying community structure characteristics. To achieve this goal, the study employed dynamic overlapping community detection methods at different k-values, which were applied to the brain network data of SZ patients and NC, respectively, to detect the respective community structures. To assess the stability and consistency of the results, the study introduced the cophenetic correlation coefficient (ccc) [38] as a metric, as shown in Equation (6). To ensure the reliability of the analyzed results, the study performed 100 independent runs and calculated the cross-run consistency between each run. In this way, the stability of the community structure under different k-values can be assessed more accurately, and guidance can be provided for selecting the optimal number of communities (k-values).

(6) $$\begin{array}{c} ccc=\frac{\sum_{i=1}^{n}\;\sum_{j=1}^{n}\;(D_{ij}-\bar{D})(C_{ij}-\bar{C})}{\sqrt{\sum_{i=1}^{n}\;\sum_{j=1}^{n}\;(D_{ij}-\bar{D}{)}^{2}\sum_{i=1}^{n}\;\sum_{j=1}^{n}\;(C_{ij}-\bar{C}{)}^{2}}} \end{array}$$

where $D_{ij}$ denotes the ith row and jth column in the original community membership matrix, and $\bar{D}$ denotes the mean value of matrix D. $C_{ij}$ denotes the ith row and jth column in the cophenetic matrix after each run of the computational hierarchical clustering tree, and $\bar{C}$ denotes the mean value of cophenetic matrix C. The cophenetic correlation coefficient measures the correlation between the original community membership matrix and the average cophenetic matrix, thus assessing the stability and consistency of the community structure.

(2) Nodes’ Functional Diversity

In complex networks, the functional diversity of nodes refers to the extent to which nodes play different roles and functions in different communities. In networks with overlapping community structures, a node can belong to multiple communities at the same time, so its functional diversity can be assessed by measuring the number of communities it participates in and the weight distribution. To quantify the functional diversity of a node, this study employed a commonly used information entropy measure, the Shannon entropy measure [39], to measure the uncertainty or diversity of a system. For a given time point i, the functional diversity of node j is calculated as shown in Equation (7):

(7) $$\begin{array}{c} H_{j}^{i}=-\sum_{p=1}^{k}\;D_{jp}^{i}lnD_{jp}^{i} \end{array}$$

where $D^{i}=C^{*}diag((z^{i}{)}^{\frac{1}{2}})$, $z^{i}=(z_{1}^{i},\ldots ,z_{k}^{i})\in R^{k}$, and $D_{jp}^{i}$ denotes the posterior probability that a node j belongs to a community p in time point i. The functional diversity of the node is highest when the brain region belongs to all communities equally, i.e., when $D_{jp}^{i}=1/k$.

(3) Activity level of overlapping communities

In order to identify the brain regions within each community and quantify their dynamics in the time domain, this study identified the brain regions within each community by setting a threshold (mean plus one standard deviation). The selection of the threshold helps to filter out brain regions that are less associated with the community, thus improving the accuracy and interpretability of the community. The rationale for choosing “mean plus one standard deviation” as the threshold is a common practice in statistics, which helps us identify brain regions that are significantly above the average and have a strong association with the community. By doing so, we can ensure that the selected brain regions have a strong functional connection with the community rather than those that fluctuate by chance or random variation. The specific activity level of overlapping communities’ representation is shown in Equation (8):

(8) $$\begin{array}{c} a_{p}=\frac{1}{\left|N_{p}\right|}z_{p}\sum_{i}\;c_{p}^{i} \end{array}$$

For each community p, all brain regions exceeding this threshold were identified as part of community p. The larger the value of the ith element in $c_{p}^{i}$ is, the greater the affiliation of the corresponding ith brain region is to the pth community. For each identified community, its dynamics in the time domain $z_{p}$ were computed, reflecting the degree of activity of community p at different points in time. $N_{p}$ denotes the number of brain regions within community p. The process involves counting the connection strengths of all brain regions within a community to capture the dynamic changes of the community.

## 3. Results

### 3.1. Structural Stability of Overlapping Communities

**Figure 2:**
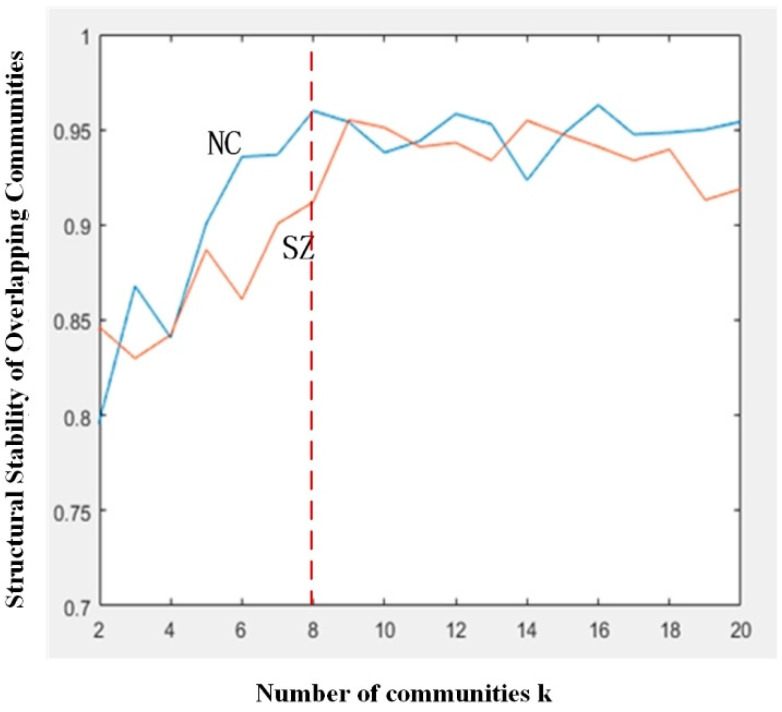
Structural stability of overlapping communities under different *k* values.

Using the overlapping community detection method, based on previous research experience, β was set to 0.1 [39]. The number of communities, *k*, was increased from 2 to 20, with an interval of 1, and for each value of *k*, the stability of the community structure was calculated, as shown in Figure 2. Figure 2 shows the cross-run concordance results for SZ patients and NC. Blue color indicates the results for the NC group, and red color indicates the results for the SZ patients. Overall, for both groups of subjects, the overall community structure stability was above 0.8. Overall, the NC group had higher community structure stability than the SZ patients. In particular, the NC group was more stable in terms of the finer community structure when the k value was larger, indicating that the NC group tended to have a more modular network structure than the SZ patients. In addition, as can be seen in Figure 2, a higher consistency of community structure was reflected in both SZ patients and NC group, where a significant change in the slope of the curve could be seen in both groups at *k* = 8. Based on the results of the curves, the k-value of both groups was set to 8 in this study.

### 3.2. Discovery of Overlapping Nodes

**Figure 3:**
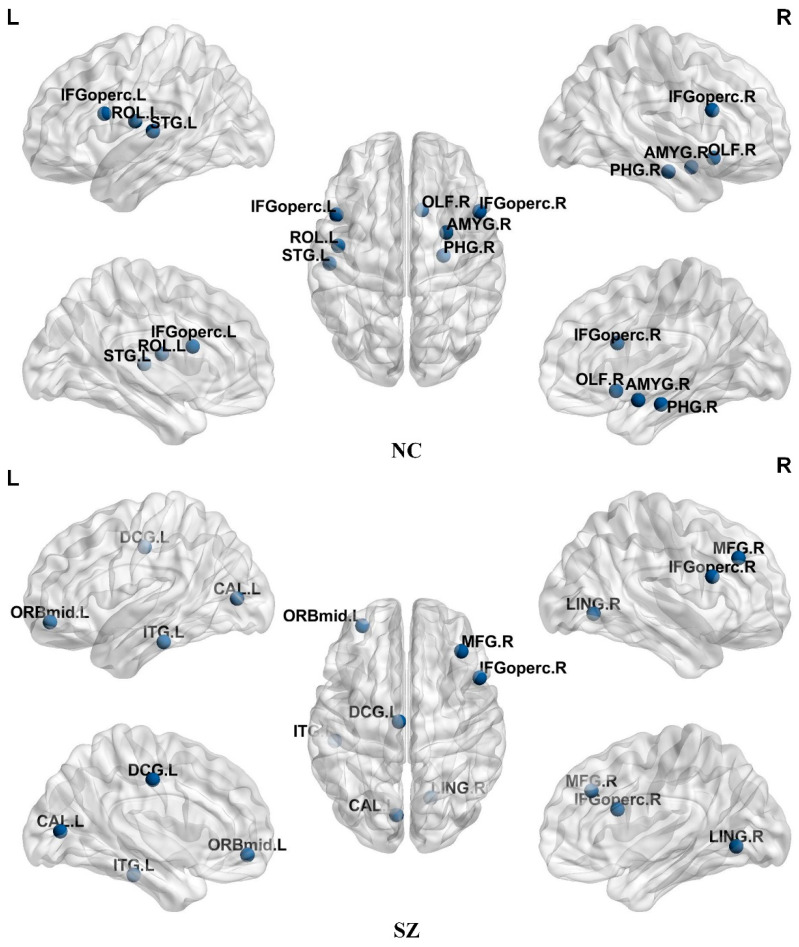
Distribution of overlapping nodes in the NC and SZ groups.

Overlapping nodes participating in multiple communities in overlapping communities were identified in this study, as shown in Figure 3 for overlapping nodes with high overlapping organization found in SZ patients and NC. The SZ patients contained seven overlapping nodes, mainly in the attention network, limbic/paralimbic and subcortical networks, and the visual network. NC contained seven overlapping nodes, mainly in the default network, attention network, limbic/paralimbic and subcortical networks, and sensorimotor network. The overlapping nodes found in the NC and SZ patients are shown in Table 2 and Table 3.

### 3.3. Nodes Functional Diversity

**Figure 4:**
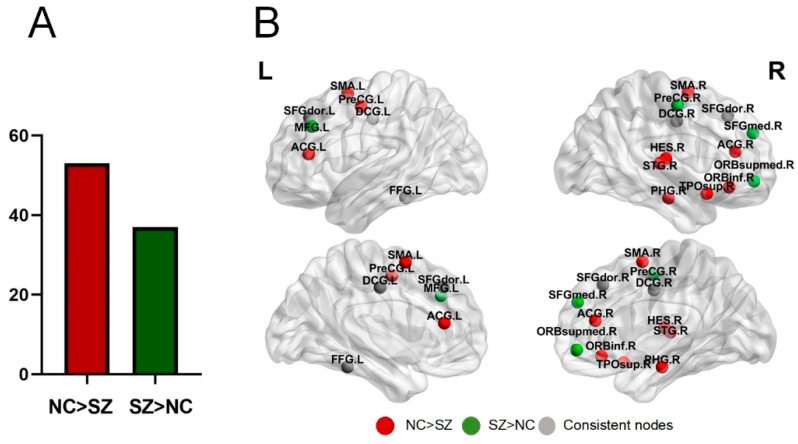
Comparison of functional diversity between nodes in the SZ group and the NC group: (**A**) the number of nodes with significant differences in functional diversity between the two groups; (**B**) the nodes with greater functional diversity in both groups.

Figure 4 demonstrates the node functional diversity results for SZ patients and NC. As shown in Figure 4A, NC had more nodes with greater functional diversity compared to SZ patients. Figure 4B demonstrates the nodes with higher functional diversity in SZ patients (green) and NC (red), where consistent nodes (gray) were those that show higher functional diversity in both SZ patients and NC.

### 3.4. Activity Level of Overlapping Communities

**Figure 5:**
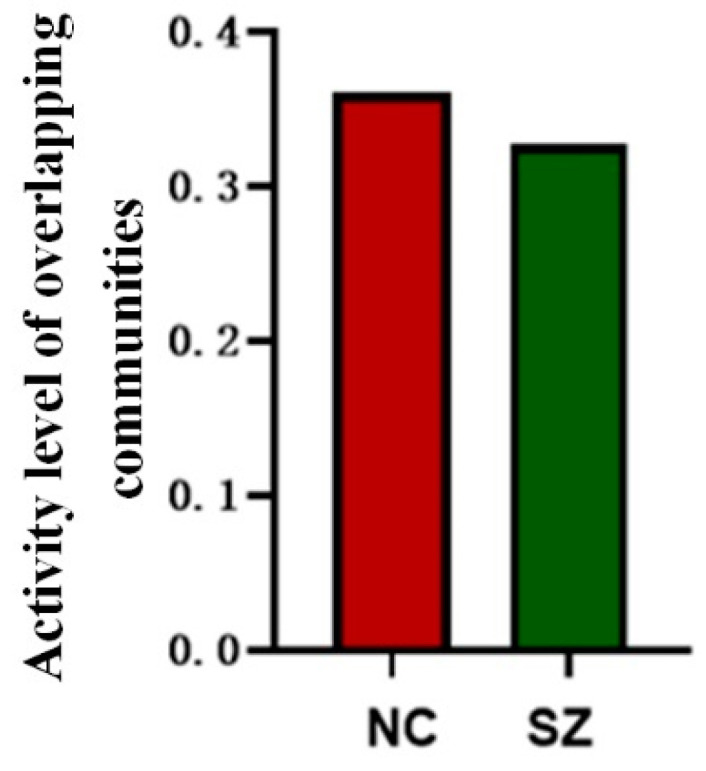
Activity level of overlapping communities.

The results of the study show that the activity level of brain communities was significantly lower in SZ patients compared to normal controls, demonstrating an unusual pattern of activity levels in SZ patients that reflects specific changes in their neural activity. A graphical representation of the relevant results is shown in Figure 5.

## 4. Discussion

### 4.1. Overlapping and Interweaving of Brain Functional Networks

In the results of this study, differences in overlapping brain regions were observed between SZ patients and NC. Specifically, the overlapping brain regions detected in SZ patients included MFG.R, ORBmid.L, IFGoperc.R, DCG.L, CAL.L, and LING.R, ITG.L, while in NC, they included IFGoperc.L, IFGoperc.R, ROL.L, OLF.R, PHG.R, AMYG.R, and STG.L. These findings demonstrate significant alterations in overlapping regions associated with brain function in SZ patients. Specifically, MFG.R and ORBmid.L are associated with cognitive control and emotion regulation, and their abnormal activity may lead to impaired attention and emotional stability in SZ patients [40]. The IFGoperc.R region plays a role in language processing and executive functioning, and its abnormal activity may lead to language deficits and decision-making difficulties in patients [41]. In addition, CAL.L and LING.R are involved in visual processing and memory functions, and abnormal activities may lead to visual perception and memory impairment [42].

The findings of this study reveal the existence of some overlapping regions of the brain that belong to multiple brain regions at the same time, providing new clues for understanding the principles of brain functional organization. The overlapping brain regions identified in our study are crucial for cognitive brain function due to their dynamic interaction between various functional modules. This interaction is modulated according to the specific demands of the task at hand, which is essential for the seamless integration and efficient transfer of information across different brain areas [43]. Past studies have shown that overlapping brain regions, which typically have a high degree of nodality and nodal efficiency, play a key role in brain networks and are critical for the transmission and integration of information [44,45].

In summary, the results of the present study demonstrate the important role of overlapping brain regions in patients with SZ, contributing to a deeper understanding of the pathophysiological mechanisms of SZ and providing new insights into the diagnosis and treatment of the disease.

### 4.2. Instability of Community Structures for SZ Patients

The aim of this study was to investigate the significance of community structural stability in the brain networks of SZ patients and its relationship with abnormal cognitive functioning. The findings revealed that SZ patients exhibited reduced stability in the community structure of their brain networks, which is of great importance for understanding the pathogenesis of the disease. We recognize that the stability of the community structure is a crucial indicator of the modularity and connectivity of brain networks. In SZ patients, this reduced stability indicates that their brain networks are more susceptible to the influence of external factors or changes in internal structure [46]. This instability can lead to abnormal functioning of the brain networks, further affecting their overall cognitive function.

A deeper analysis shows that the instability of the community structure in SZ patients is closely related to abnormal cognitive functioning. Due to confusion and disorganization in information processing, patients’ cognitive abilities, emotional regulation, and daily functioning are all impacted. Moreover, this low stability suggests that brain regions are unable to efficiently integrate multimodal information on long time scales, indicating significant changes in the functional and connectivity patterns of the brain network at different points in time or under different conditions, leading to fragmented and unstable information processing. Compared with previous studies, our findings further confirm the importance of brain network structure stability in mental disorders. Prior research has already pointed out that abnormal connections and dysfunctional brain networks are closely related to cognitive and behavioral symptoms in SZ and other mental illnesses [47]. However, this study specifically focuses on the link between community structural stability and abnormal cognitive functioning, providing a new perspective for understanding the pathogenesis of mental disorders.

In conclusion, this study reveals the close relationship between reduced community structural stability and abnormal cognitive functioning in SZ patients. This instability not only impairs the brain’s ability to process and integrate information but also leads to cognitive decline, disorganized information processing, and maladaptation to external stimuli. Therefore, an in-depth study of the association between community structural instability and abnormal cognitive functioning will help us better understand the pathogenesis of mental disorders and provide new ideas and methods for the diagnosis and treatment of related disorders.

### 4.3. Reduction in Community Activity Level and Functional Diversity of Nodes in SZ Patients

Community activity level reflects the activity and functional status of different brain regions in the brain network, while a low community activity level indicates impaired or abnormal brain function in SZ patients [48]. Reduced activity level of overlapping communities reflects the incoherence of brain network function in SZ patients. Specifically, a reduced activity level of overlapping communities is detrimental to information transfer within the brain network and affects the normal functioning of cognitive and emotional processing.

In addition, this study observed that the functional diversity of nodes was generally lower in SZ patients than in NC, indicating an impaired functional diversity of brain networks in SZ patients, reflecting the abnormal state of brain function in patients. Reduced functional diversity implies that different regions in the patient’s brain are more homogeneous or similar in their functional performance, lacking the original diversity and flexibility. This condition may lead to limitations in information processing and integration of brain networks, which, in turn, affects patients’ cognitive, emotional, and behavioral functions.

In this study, we focused on abnormal nodes with a low functional diversity of nodes in SZ patients. Specifically, regions with low node functional diversity in SZ patients include areas involved in a wide range of different cognitive and emotional functions. For example, the supplementary motor area, which is located before the primary motor areas in the left and right hemispheres of the brain, is a premotor area [49]. It is involved in planning, organizing, and integrating information and is responsible for the initial stages of the movement. The supplementary motor area is part of a larger brain network that mediates information processing in a variety of cognitive functions. Yuan et al. found that activity in the posterior portion of the supplementary motor area was eliminated or reduced in patients with SZ [40]. It was shown that these brain regions are involved in speech and language processing, including motor planning and control, as well as attentional switching and inner speech during language encoding. In general, most of these brain regions belong to the anatomical models of auditory language, speech production, and reading. Reduced nodal functional diversity means that this brain region has a diminished ability to assume complex and diverse roles in multiple functional networks. This finding is consistent with findings of reduced community vitality in patients with schizophrenia. These results further highlight the importance of abnormal brain network functioning in SZ patients and provide key clues for a deeper understanding of the pathophysiological mechanisms of this disorder as well as for the development of new therapeutic strategies [50].

## 5. Limitations and Directions for Future Research

While our study provides valuable insights, it also has some limitations. The sample size may limit the generalizability of our results, and future studies should consider a larger sample size to enhance the reliability of the conclusions. Additionally, potential selection bias or information bias may affect the interpretation of the results. Future research should address these issues by employing more diverse samples and stricter data collection methods.

Future research should further explore the clinical applications of these findings, especially in the context of personalized medicine and early intervention. Moreover, future studies should consider employing more advanced neuroimaging techniques, such as functional magnetic resonance imaging (fMRI) and positron emission tomography (PET), to gain a deeper understanding of the changes in the brain networks of SZ patients. Through these methods, we can more comprehensively understand the pathophysiological mechanisms of SZ and develop more effective treatment strategies.

## 6. Conclusions

This study employed a matrix factorization-based dynamic overlapping community detection method to delve into the aberrant overlapping community structure in the brains of patients with SZ. Our findings revealed a significant decline in the stability of SZ patients’ brain functional networks, indicating potential anomalies or disruptions in the structure or organization of their cerebral connectivity. Moreover, the observed changes in the overlapping community structure of SZ patients were associated with a reduction in the diversity of node functions, reflecting alterations in neural connectivity within the pathological process of SZ. Compared to NC, a marked decrease in community activity level in SZ patients further confirmed the impaired functionality of their brain networks in information processing and transmission. These discoveries offer new insights into the pathogenesis of SZ, highlighting the potential of dynamic network analysis in uncovering abnormalities in brain connectivity. The results of our study not only enhance the understanding of the pathophysiological mechanisms of SZ but also may provide crucial biomarkers for future diagnostic, therapeutic, and intervention strategies. By building on these findings, we hope to develop treatments targeting the abnormal network activities, aiming to improve the clinical symptoms and quality of life for individuals with SZ.

## Figures and Tables

**Table 1:** Demographic and clinical characteristics in this study.

Characteristic	SZ	NC	Statistical Test
Number of subjects	43	49	–
Age (years)	34.84 ± 8.60	33.12 ± 8.22	*p* = 0.331
Sex (male/female)	30/13	30/19	*p* = 0.112

Note: the values are denoted as mean ± standard deviation.

**Table 2:** Overlapping nodes in NC.

Full Name of the Brain Region	Brain Area Abbreviations	ROI	RSN
Frontal_Inf_Oper_L	IFGoperc.L	11	Attention Network
Frontal_Inf_Oper_R	IFGoperc.R	12	Attention Network
Rolandic_Oper_L	ROL.L	17	Sensorimotor Network
Olfactory_R	OLF.R	22	Default Network
ParaHippocampal_R	PHG.R	40	Limbic/paralimbic and Subcortical Networks
Amygdala_R	AMYG.R	42	Limbic/paralimbic and Subcortical Networks
Temporal_Sup_L	STG.L	81	Sensorimotor Network

**Table 3:** Overlapping nodes in SZ patients.

Full Name of the Brain Region	Brain Area Abbreviations	ROI	RSN
Frontal_Mid_R	MFG.R	8	Attention Network
Frontal_Mid_Orb_L	ORBmid.L	9	Attention Network
Frontal_Inf_Oper_R	IFGoperc.R	12	Attention Network
Cingulum_Mid_L	DCG.L	33	Limbic/paralimbic and Subcortical Networks
Calcarine_L	CAL.L	43	Visual Network
Lingual_R	LING.R	48	Visual Network
Temporal_Inf_L	ITG.L	89	Attention Network

## Data Availability

The data presented in this study are openly available in [openfMRI] at [https://openfmri.org/dataset/ds000030/ (accessed on 18 October 2021)].

## Appendix A

**Table A1:** Mapping between ROI names and experiential function networks in AAL template.

ROI	Regions	Abbreviated	RSN
1	Precentral_L	PreCG.L	DMN
2	Precentral_R	PreCG.R	DMN
3	Frontal_Sup_L	SFGdor.L	DMN
4	Frontal_Sup_R	SFGdor.R	DMN
5	Frontal_Sup_Orb_L	ORBsup.L	DMN
6	Frontal_Sup_Orb_R	ORBsup.R	DMN
7	Frontal_Mid_L	MFG.L	Attention
8	Frontal_Mid_R	MFG.R	Attention
9	Frontal_Mid_Orb_L	ORBmid.L	Attention
10	Frontal_Mid_Orb_R	ORBmid.R	Attention
11	Frontal_Inf_Oper_L	IFGoperc.L	Attention
12	Frontal_Inf_Oper_R	IFGoperc.R	Attention
13	Frontal_Inf_Tri_L	IFGtriang.L	Attention
14	Frontal_Inf_Tri_R	IFGtriang.R	Attention
15	Frontal_Inf_Orb_L	ORBinf.L	Attention
16	Frontal_Inf_Orb_R	ORBinf.R	Attention
17	Rolandic_Oper_L	ROL.L	Sensorimotor
18	Rolandic_Oper_R	ROL.R	Sensorimotor
19	Supp_Motor_Area_L	SMA.L	Attention
20	Supp_Motor_Area_R	SMA.R	Sensorimotor
21	Olfactory_L	OLF.L	Subcortical
22	Olfactory_R	OLF.R	DMN
23	Frontal_Sup_Medial_L	SFGmed.L	DMN
24	Frontal_Sup_Medial_R	SFGmed.R	DMN
25	Frontal_Mid_Orb_L	ORBsupmed.L	DMN
26	Frontal_Mid_Orb_R	ORBsupmed.R	DMN
27	Rectus_L	REC.L	DMN
28	Rectus_R	REC.R	DMN
29	Insula_L	INS.L	Sensorimotor
30	Insula_R	INS.R	Sensorimotor
31	Cingulum_Ant_L	ACG.L	DMN
32	Cingulum_Ant_R	ACG.R	DMN
33	Cingulum_Mid_L	DCG.L	Subcortical
34	Cingulum_Mid_R	DCG.R	Subcortical
35	Cingulum_Post_L	PCG.L	DMN
36	Cingulum_Post_R	PCG.R	DMN
37	Hippocampus_L	HIP.L	Subcortical
38	Hippocampus_R	HIP.R	Subcortical
39	ParaHippocampal_L	PHG.L	Subcortical
40	ParaHippocampal_R	PHG.R	Subcortical
41	Amygdala_L	AMYG.L	Subcortical
42	Amygdala_R	AMYG.R	Subcortical
43	Calcarine_L	CAL.L	Visual
44	Calcarine_R	CAL.R	Visual
45	Cuneus_L	CUN.L	Visual
46	Cuneus_R	CUN.R	Visual
47	Lingual_L	LING.L	Visual
48	Lingual_R	LING.R	Visual
49	Occipital_Sup_L	SOG.L	Visual
50	Occipital_Sup_R	SOG.R	Visual
51	Occipital_Mid_L	MOG.L	Visual
52	Occipital_Mid_R	MOG.R	Visual
53	Occipital_Inf_L	IOG.L	Visual
54	Occipital_Inf_R	IOG.R	Visual
55	Fusiform_L	FFG.L	Visual
56	Fusiform_R	FFG.R	Visual
57	Postcentral_L	PoCG.L	Sensorimotor
58	Postcentral_R	PoCG.R	Sensorimotor
59	Parietal_Sup_L	SPG.L	Sensorimotor
60	Parietal_Sup_R	SPG.R	Sensorimotor
61	Parietal_Inf_L	IPL.L	Attention
62	Parietal_Inf_R	IPL.R	Attention
63	SupraMarginal_L	SMG.L	Sensorimotor
64	SupraMarginal_R	SMG.R	Sensorimotor
65	Angular_L	ANG.L	Attention
66	Angular_R	ANG.R	Attention
67	Precuneus_L	PCUN.L	DMN
68	Precuneus_R	PCUN.R	DMN
69	Paracentral_Lobule_L	PCL.L	Sensorimotor
70	Paracentral_Lobule_R	PCL.R	Sensorimotor
71	Caudate_L	CAU.L	Subcortical
72	Caudate_R	CAU.R	Subcortical
73	Putamen_L	PUT.L	Subcortical
74	Putamen_R	PUT.R	Subcortical
75	Pallidum_L	PAL.L	Subcortical
76	Pallidum_R	PAL.R	Subcortical
77	Thalamus_L	THA.L	Subcortical
78	Thalamus_R	THA.R	Subcortical
79	Heschl_L	HES.L	Sensorimotor
80	Heschl_R	HES.R	Sensorimotor
81	Temporal_Sup_L	STG.L	Sensorimotor
82	Temporal_Sup_R	STG.R	Sensorimotor
83	Temporal_Pole_Sup_L	TPOsup.L	Attention
84	Temporal_Pole_Sup_R	TPOsup.R	Sensorimotor
85	Temporal_Mid_L	MTG.L	DMN
86	Temporal_Mid_R	MTG.R	DMN
87	Temporal_Pole_Mid_L	TPOmid.L	Subcortical
88	Temporal_Pole_Mid_R	TPOmid.R	Subcortical
89	Temporal_Inf_L	ITG.L	Attention
90	Temporal_Inf_R	ITG.R	DMN
